# Assessing patients’ risk of febrile neutropenia: is there a correlation between physician-assessed risk and model-predicted risk?

**DOI:** 10.1002/cam4.454

**Published:** 2015-03-23

**Authors:** Gary H Lyman, David C Dale, Jason C Legg, Esteban Abella, Phuong Khanh Morrow, Sadie Whittaker, Jeffrey Crawford

**Affiliations:** 1 Hutchinson Institute for Cancer Outcomes Research, Fred Hutchinson Cancer Research CenterSeattle, Washington; 2 Department of Medicine, University of WashingtonSeattle, Washington; 3 Global Biostatistical Science, Amgen Inc.Thousand Oaks, California; 4 Hematology/Oncology, Amgen Inc.Thousand Oaks, California; 5 Department of Medicine, Duke University School of Medicine and Duke Cancer InstituteDurham, North Carolina

**Keywords:** Chemotherapy, febrile neutropenia, granulocyte colony-stimulating factor, neutropenia, primary prophylaxis, risk assessment, risk factors, risk model, severe neutropenia

## Abstract

This study evaluated the correlation between the risk of febrile neutropenia (FN) estimated by physicians and the risk of severe neutropenia or FN predicted by a validated multivariate model in patients with nonmyeloid malignancies receiving chemotherapy. Before patient enrollment, physician and site characteristics were recorded, and physicians self-reported the FN risk at which they would typically consider granulocyte colony-stimulating factor (G-CSF) primary prophylaxis (FN risk intervention threshold). For each patient, physicians electronically recorded their estimated FN risk, orders for G-CSF primary prophylaxis (yes/no), and patient characteristics for model predictions. Correlations between physician-assessed FN risk and model-predicted risk (primary endpoints) and between physician-assessed FN risk and G-CSF orders were calculated. Overall, 124 community-based oncologists registered; 944 patients initiating chemotherapy with intermediate FN risk enrolled. Median physician-assessed FN risk over all chemotherapy cycles was 20.0%, and median model-predicted risk was 17.9%; the correlation was 0.249 (95% CI, 0.179−0.316). The correlation between physician-assessed FN risk and subsequent orders for G-CSF primary prophylaxis (*n* = 634) was 0.313 (95% CI, 0.135−0.472). Among patients with a physician-assessed FN risk ≥20%, 14% did not receive G-CSF orders. G-CSF was not ordered for 16% of patients at or above their physician’s self-reported FN risk intervention threshold (median, 20.0%) and was ordered for 21% below the threshold. Physician-assessed FN risk and model-predicted risk correlated weakly; however, there was moderate correlation between physician-assessed FN risk and orders for G-CSF primary prophylaxis. Further research and education on FN risk factors and appropriate G-CSF use are needed.

## Introduction

Febrile neutropenia (FN) following myelosuppressive chemotherapy is associated with substantial mortality and costs 1,2. Current guidelines recommend primary prophylaxis with colony-stimulating factors (CSFs), such as granulocyte colony-stimulating factor (G-CSF), when the FN risk is 20% or greater 3,4. Individual FN risk depends on disease-specific factors (e.g., tumor type), patient-specific factors (e.g., comorbidities), and treatment-specific factors (e.g., type and intensity of chemotherapy) 5–11. Thus, precise and consistent FN risk assessment is essential.

To identify patients likely to benefit from G-CSF prophylaxis, a multivariate model for predicting the risk of severe neutropenia (SN) or FN during chemotherapy cycle 1 was developed and validated using a large, prospective registry of patients receiving myelosuppressive chemotherapy for nonmyeloid cancer 12. SN events were included because they are more frequent than FN events and are not susceptible to individual variations in antibiotics use and response. Cycle 1 was chosen because that is when FN most frequently occurs^5^,^6^,^9^,^13^ and is when patients are more likely to receive a full chemotherapy dose relative to subsequent cycles; after cycle 1, FN risk may decrease due to chemotherapy dose reductions/delays, G-CSF secondary prophylaxis, and/or antibiotic use. A high concordance was observed between the model-predicted and actual SN or FN risk 12. Furthermore, a strong association was observed between the predicted SN or FN risk during cycle 1 and the actual FN risk in cycles 1–4.

The primary objective of the current multicenter observational study was to investigate the correlation between physician-assessed FN risk and model-predicted risk in patients with nonmyeloid malignancies. The secondary objective was to assess the correlation between physician-assessed FN risk and subsequent orders for primary prophylaxis with G-CSF.

## Patients and Methods

### Patients

Eligible patients included adults with any stage non-Hodgkin’s lymphoma or small cell lung, non-small cell lung, ovarian, colorectal, or breast cancer initiating a new standard-dose chemotherapy regimen (±15% on any component) that was associated with an intermediate FN risk (10−20% at first use based on the regimen alone) per the National Comprehensive Cancer Network (NCCN; Table S1). Exclusion criteria included receiving any chemotherapy regimen with a cycle length ≤12 days; prior stem cell or bone marrow transplantation; current enrollment in a clinical trial requiring CSFs or a clinical trial on an investigational device or drug, or <30 days since ending participation in a clinical trial on an investigational device or drug; and receiving chemotherapy for palliation or with planned cycle 1 chemotherapy dose reduction >15%. The study protocol was approved by the institutional review boards at each site and patients provided written informed consent. Patients from each practice were screened by site staff for eligibility and subsequently clinically evaluated by their physician for eligibility for the study.

### Study design and procedures

**Figure 1:**
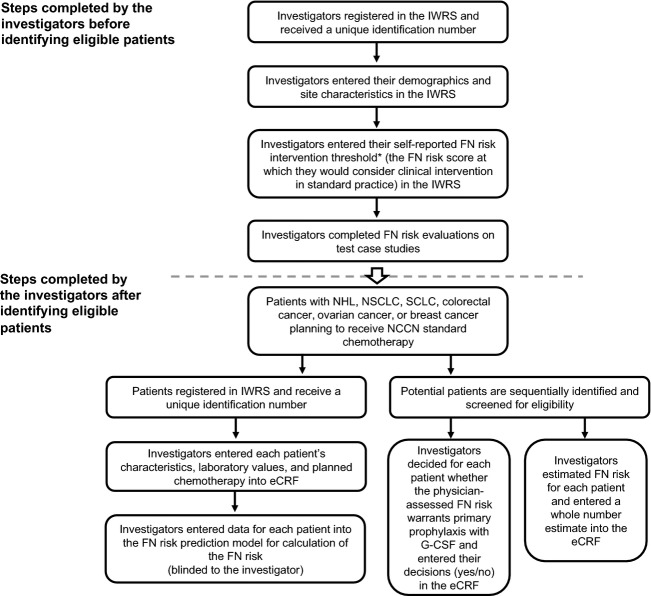
Study design schema. eCRF, electronic case report form; FN, febrile neutropenia; G-CSF, granulocyte colony-stimulating factor; IWRS, interactive web response system; NCCN, National Comprehensive Cancer Network; NHL, non-Hodgkin’s lymphoma; NSCLC, non-small cell lung cancer; SCLC, small cell lung cancer.*The physician’s self-reported FN risk intervention threshold was entered as a whole number; once entered, it could not be changed..

This observational study was conducted at 56 centers in the United States. Study sites and physicians were recruited based on interest surveys and feasibility of recruiting patients based on the inclusion and exclusion criteria. Participating physicians were compensated at a standard rate for their and/or their study coordinators’ time. Prior to identifying patients, physicians were required to register in the interactive web response system and enter their demographics, site characteristics, and self-reported FN risk intervention threshold (the physician-assessed FN risk estimates at or above which the physician would typically consider G-CSF primary prophylaxis). Physicians also completed FN risk evaluations on four hypothetical test case studies for measurement of interphysician variability in rating common cases. Physicians followed a specific procedure for the enrollment of patients and the collection and entry of physician, site, and patient information (Fig.1). To reduce the influence of any individual physician or site on the analysis, the number of investigators per site was limited to four, and the number of patients per investigator was limited to 14. Physicians screened sequential patients for eligibility in an effort to minimize selection bias. For each screened patient, the patient’s physician estimated the FN risk based on routine clinical practice, entered it into the electronic case report form (eCRF), and recorded whether the patient received orders for primary prophylaxis with G-CSF (yes/no). For each registered patient, the patient’s physician entered characteristics, laboratory values, and planned chemotherapy into the eCRF for generation of the prediction model SN or FN risk. Physicians were blinded to the data elements used in the model and the resultant prediction. The study concluded when the model prediction was complete and orders for the first cycle of chemotherapy and G-CSF were written. No patient outcome data were collected, including administration of chemotherapy and occurrences of FN.

### Statistical analysis

In the original model derivation and validation study, a strong association was observed between the predicted SN or FN risk during chemotherapy cycle 1 and the actual FN risk in cycles 1–4 (Fig. S1). The primary endpoints in the current study were the physician-assessed FN risk and the model-predicted risk. The secondary endpoints were physician self-reported FN risk intervention threshold and orders for prophylactic G-CSF in chemotherapy cycle 1 (before day 4).

The study sample size was based on specification of the anticipated confidence interval width for the correlation between physician-assessed FN risk and model-predicted risk (see Data S1). To allow some investigators to enroll <14 patients and to allow for possible protocol deviations, the planned sample size was 1000 patients distributed among 80 investigators in community-based oncology practices. To achieve adequate patient enrollment, a greater number of investigators was recruited than initially planned. Patients who met all inclusion criteria and had a physician-assessed FN risk and model-predicted risk comprised the primary analysis set, whereas investigators who enrolled patients in the study comprised the investigator analysis set.

Correlations were calculated using the Pearson correlation coefficient. Variance was estimated using the delete-a-physician jackknife variance estimator (see Data S1) 14. Confidence intervals for the correlation coefficient were constructed using the Fisher transformation procedure 15,16. The relationship between physician-assessed FN risk and model-predicted risk was estimated using penalized splines with a cluster bootstrap to produce pointwise confidence limits 17. The proportion of patients with a physician-assessed FN risk above the investigator’s intervention threshold and who received an order for G-CSF was represented as a standard proportion and a Wald confidence interval using the jackknife variance estimator. Investigator demographics, patient demographics, and disease characteristics were summarized using descriptive statistics. Patients with missing physician-assessed FN risk estimates or data necessary for calculation of model-predicted risk were excluded from the analysis (Fig. S2). Potential bias from measurement errors was mitigated by reviewing laboratory measurements for outliers and reviewing physician-assessed risk estimates, risk thresholds, and G-CSF orders against physician comments provided in the eCRF.

## Results

### Physician and patient characteristics

Between 16 June 2011 and 16 November 2012, 124 physicians registered for this study (Table1). The median length of clinical experience was 12 years; 64% of physicians practiced in a small clinic setting, and 80% were self-described hematologists/oncologists. The median physician self-reported FN risk intervention threshold was 20.0% (range, 5−60%).

**Table 1:** Physician Demographics^1^

	Investigators (*N* = 124)
Median (range) time in clinical practice, years	12.0 (1−35)
Median (range) patients treated in clinic per month, *n*	350 (12−999)
Primary specialty, *n* (%)	
Hematologist/oncologist	99 (80)
Oncologist	23 (19)
Gynecologist/oncologist	2 (2)
Type of clinical practice, *n* (%)	
Single specialty	82 (66)
Multiple subspecialties	42 (34)
Clinical setting, *n* (%)	
≤4 physicians	79 (64)
>4 physicians	45 (36)
Median (Q1−Q3) self-reported FN risk intervention threshold across all chemotherapy cycles^2^, %	20.0 (15.0–20.0)

FN, febrile neutropenia.

1 Investigator analysis set.

2 The FN risk at which the investigator would consider ordering G-CSF in usual standard practice.

Forty-two of 986 enrolled patients were excluded because of failure to meet eligibility criteria (*n* = 35), missing data (*n* = 4), and enrollment errors (*n* = 3); therefore, 944 patients were included in the primary analysis set (Fig. S2). Women outnumbered men (*n* = 618 vs. *n* = 326) in the primary analysis set, reflecting the high frequency (39%) of breast cancer (Table2). Other tumor types included colorectal (27%), non-small cell lung (12%), non-Hodgkin’s lymphoma (11%), small cell lung (9%), and ovarian (2%) cancer. The majority of patients had advanced disease (stage III, 25%; stage IV, 31%). The prevalence of diabetes and hypertension was 20% and 50%, respectively, which is slightly higher than in adults in the general population 18,19. Patient baseline laboratory test results are shown in Table S2. Planned chemotherapy regimens of interest were cyclophosphamide and docetaxel (TC; *n* = 198), carboplatin, docetaxel, and trastuzumab (TCH; *n* = 83), cyclophosphamide and doxorubicin (AC; *n* = 49), and AC + sequential taxane ± trastuzumab (AC + T; *n* = 22) for patients with breast cancer; fluorouracil, leucovorin, and oxaliplatin (FOLFOX; *n* = 218) for patients with colorectal cancer; and cyclophosphamide, doxorubicin, vincristine, and prednisone (CHOP-based; *n* = 101) for patients with non-Hodgkin’s lymphoma.

**Table 2:** Patient demographics, disease characteristics, and comorbidities^1^

	Patients (*N* = 944)
Sex, *n* (%)	
Men	326 (35)
Women	618 (66)
Median (range) age, years	62 (23−90)
Age group, *n* (%)	
<65 years	553 (59)
≥65 years	391 (41)
Mean (SD) body mass index, kg/m^2^	28.8 (6.7)
Tumor type	
Breast	364 (39)
Colorectal	259 (27)
Non-small cell lung	115 (12)
Non-Hodgkin’s lymphoma	106 (11)
Small cell lung	83 (9)
Ovarian	17 (2)
Disease stage	
I	129 (14)
II	201 (21)
III	238 (25)
IV	294 (31)
Not available	82 (9)
ECOG performance status	
0	545 (58)
1	294 (31)
2	57 (6)
>2	11 (1)
Missing	37 (4)
Prior chemotherapy	141 (15)
Planned use of immunosuppressives	10 (1)
Comorbidities	
High blood pressure	472 (50)
Diabetes	190 (20)
COPD/pulmonary disease	135 (14)
Kidney disease	59 (6)
Autoimmune disease	36 (4)
Liver dysfunction	33 (4)
Congestive heart failure	18 (2)
HIV positive	2 (<1)

COPD, chronic obstructive pulmonary disease; Eastern Cooperative Oncology Group.

1 Primary analysis set.

### Correlation between physician-assessed FN risk and model-predicted risk estimates

**Figure 2:**
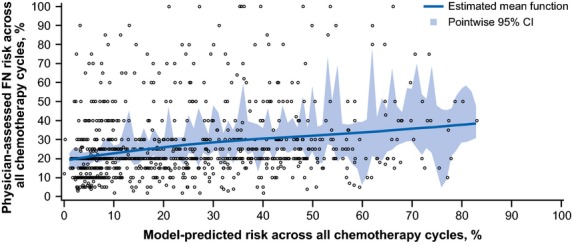
Spline fit for the correlation between the physician-assessed and model-predicted febrile neutropenia risk over all chemotherapy cycles. The line indicates the estimated mean function, and the shaded area indicates the 95% pointwise CI. Physician-assessed FN risk estimates varied considerably around the model-predicted risk estimates and did not increase linearly with increasing model-predicted estimates above 30%. FN, febrile neutropenia.

In the primary analysis, the median physician-assessed FN risk over all chemotherapy cycles was 20.0% (Q1−Q3, 15−30%), and the median model-predicted risk in cycle 1 was 17.9% (Q1−Q3, 6.9−35.8%; Table3). A weak correlation (0.249; 95% CI, 0.179−0.316) was observed between the physician-assessed FN risk estimate and the model-assessed risk estimate using the Pearson correlation coefficient (Fig.2). As the model-predicted risk estimates increased beyond 30%, the relationship between the model and physician-assessed risk estimates of FN became less linear as seen by the decreased slope of the fit line. Further analysis demonstrated equivalent or greater correlation between the physician-assessed FN risk and model-predicted risk estimates by certain patient-, physician-, and site-related characteristics, such as patient age <65 years (0.263), planned TCH regimen (0.254), physician with between 8 and 17 years of clinical practice experience (0.306), oncology as primary specialty (0.286), and practice treating ≤212 patients per month (first tertile; 0.306), and tumor type (0.249; Table S3).

**Table 3:** Summary of physician-assessed risk FN estimates, model-predicted risk estimates, and G-CSF orders^1^

	Patients (*N* = 944)
Median (Q1−Q3) physician-assessed FN risk estimate over all chemotherapy cycles, %	20.0 (15.0–30.0)
Median (Q1−Q3) model-predicted risk estimate, %	17.9 (6.9−35.8)
Correlation estimate (approximate 95% CI^2^)	0.249^3^ (0.179−0.316)
Order for primary prophylaxis with G-CSF, *n* (%)	634 (67)
Correlation estimate (approximate 95% CI^2^)	0.313^3^ (0.135−0.472)

FN, febrile neutropenia; G-CSF, granulocyte colony-stimulating factor; SN, severe neutropenia.

1 Primary analysis set.

2 Confidence interval calculated using the cluster jackknife estimator and Wald method with Fisher transformation.

3 Correlations can range from 1 (perfect correlation) to −1, where 0 is no correlation, and negative correlations represent inverse relationships.

### Correlation between physician-assessed risk estimates and G–CSF orders

Among all patients (*n* = 944), physician-assessed FN risk estimates over all chemotherapy cycles correlated moderately with subsequent G-CSF orders using the Pearson correlation coefficient (0.313; 95% CI, 0.135−0.472; Table3). Further analysis demonstrated greater correlation between physician-assessed FN risk estimates and subsequent G-CSF orders by certain patient-, physician-, and site-related characteristics, such as colorectal tumors (0.514), planned FOLFOX regimen (0.478), physician with >8–17 years of clinical practice experience (0.451), oncology as primary specialty (0.452), practice treating ≤212 patients per month (first tertile; 0.477), and practice with >4 physicians (0.422; Table S4).

Overall, 634 of 944 patients (67%) received orders for G-CSF (Table3). Among these, the median physician-assessed FN risk over all chemotherapy cycles was 25.0% (Q1−Q3, 20.0−35.0%), and the median model-predicted risk was 22.2% (9.1−39.7%; correlation, 0.172; 95% CI, 0.088−0.254; Table S5). Of the 310 patients (33%) who did not receive an order for G-CSF, the median physician-assessed FN risk over all chemotherapy cycles was 15.0% (Q1−Q3, 10.0−20.0%), and the median model-predicted risk was 8.5% (4.1−24.8%; correlation, 0.239; 95% CI, 0.000−0.453).

A total of 637 of 944 patients (67%) had a physician-assessed FN risk of ≥20%, 550 (86%) of whom received orders for G-CSF. Notably, 117 of 944 patients (12%) had a physician-assessed FN risk ≥50%, 101 (86%) of whom received orders for G-CSF.

Overall, 692 of 944 patients (73%) had a physician-assessed FN risk at or above the physicians’ self-reported risk intervention threshold. Among these, 582 (84%) received an order for primary prophylaxis with G-CSF. Of the 252 patients (27%) with a physician-assessed FN risk below the physicians’ risk intervention threshold, 52 (21%) received an order for primary prophylaxis with G-CSF.

## Discussion

Given the variety of patient-, disease-, and treatment-related factors that may increase FN risk among patients receiving myelosuppressive chemotherapy regimens 5–10,20–22, a standardized and systematic approach to predicting FN risk could facilitate appropriate use of G-CSFs 12,20. Lyman and colleagues previously developed and validated a multivariate model to predict the SN or FN risk in patients initiating chemotherapy 12. In the multicenter observational study reported here, physician-assessed FN risk over all chemotherapy cycles correlated weakly with model-predicted risk in patients initiating chemotherapy with an intermediate FN risk based on NCCN guidelines. The median values for physician-assessed FN risk and model-predicted risk were similar, but there were sizeable differences between individual pairs of physician-assessed and model-predicted risk estimates. Physician-assessed FN risk estimates varied widely and did not consistently increase as model-predicted risk estimates increased beyond 30%, suggesting that the physicians included in this study may have underestimated FN risk for some regimen and patient characteristic combinations.

The weak correlation between physician-assessed FN risk and model-predicted risk may reflect variation in several factors, including differences in how the physicians weighted the importance of the patient-, disease-, and treatment-related risk factors included in the multivariate model (e.g., previous chemotherapy, abnormal hepatic or renal function, reduced white blood cell count, chemotherapy, and planned relative dose intensity ≥85%) 12. The correlation of the physician-assessed FN risk and model-predicted risk may have also been influenced by differences in physician and site characteristics (e.g., years in practice and number of patients treated), as well as physicians’ tendency to provide FN risk estimates in multiples of 5%, rather than more precise estimates. Furthermore, variation in severity of neutropenia and the timeframe (i.e., all chemotherapy cycles versus cycle 1) between the physician-assessed FN risk and the model-predicted risk estimates may have reduced the correlation.

Primary prophylaxis with CSFs is recommended for patients whose FN risk is ≥20%, with consideration of patient-specific risk factors and the intent of treatment (e.g., curative versus palliation) 3,4,23,24. In this study, physician-assessed FN risk estimates over all chemotherapy cycles correlated moderately with subsequent G-CSF orders. Although the majority (86%) of patients who had a physician-assessed FN risk ≥20% received an order for G-CSF primary prophylaxis, 14% did not. Interestingly, the same proportion (14%) of patients who had a physician-assessed FN risk ≥50% did not receive an order for G-CSF primary prophylaxis, despite their elevated risk. In the present study, consideration of patient-specific factors (e.g., planned chemotherapy) may have contributed to a physician decision against G-CSF primary prophylaxis in some patients with elevated FN risk. However, the results indicate a need for physicians to improve alignment of FN risk assessment and appropriate G-CSF use. Among patients above the physicians’ self-reported FN risk intervention threshold (those who would typically require G-CSF), 16% did not receive G-CSF orders. Among patients below this threshold (those who would typically not require G-CSF), 21% received G-CSF orders. Collectively, these results suggest that continued education for physicians regarding FN risk factors, guidelines, and appropriate use of G-CSF primary prophylaxis is needed in both the US and Europe 25,26. Additional research could help clarify the clinical circumstances in which G-CSF orders do not align with guidelines for FN management.

This observational study had potential limitations related to site and physician selection, physician behavior, study design, and changes over time in the understanding of treatment-specific risk factors. Study sites and physicians were recruited using interest surveys and based on feasibility of the site to recruit patients. This nonrandom selection of sites, physicians within sites, and patients by physicians had the potential to introduce bias. The practices of some physicians may have been overrepresented relative to the target population, and bias may have been introduced via errors in laboratory measurements and variations in FN risk assessments among physicians. Physician behavior may have been altered because of the need to identify and record risk factors, report risks, and disclose orders for G-CSF. Although some physicians provided their rationale for ordering G-CSF for patients below their FN risk intervention threshold and/or for not ordering G-CSF for patients above their intervention threshold, responses were recorded in a free-text format and varied widely; no clear trends were observed, limiting the utility of these responses. This study was designed to evaluate the correlation between physician-assessed FN risk and model-predicted risk; the actual incidence of FN was not captured. A high concordance between actual FN risk and model-predicted risk was observed in a large prospective patient population 12; however, the model has not been independently validated. Thus, the model is used here as a comparative benchmark to evaluate physician risk assessment and decision making, not as a replacement for physician decision making. This pilot study was too small to determine definitive quantitative relationships among physician-assessed FN risk, G-CSF ordering patterns, and physician- and patient-related factors. Finally, the study may also have been limited by change over time in the FN risk attributed to TC, which was initially considered to have a low-to-intermediate FN risk but was subsequently reported to have high FN risk (>20%) 11,27. TC was planned for the majority of patients with breast cancer—the most frequent cancer type in this study.

In conclusion, this study demonstrated the feasibility of studying healthcare delivery, with respect to FN assessment and G-CSF orders, at the individual physician and practice level. Our analysis revealed a weak correlation between physician-assessed FN risk and model-predicted risk and a moderate correlation between physician-assessed FN risk and subsequent orders for G-CSF. These findings illustrate the heterogeneity of physicians’ assessment of FN risk and utilization of G-CSF. Hence, further research and education on the risk factors for FN, guidelines for FN management, and appropriate G-CSF primary prophylaxis are needed to optimize supportive care of patients most at risk for neutropenic complications. The results of this small pilot study indicate that additional larger prospective studies of factors that affect physicians’ FN risk assessment and appropriate utilization of G-CSF in support of patients with cancer receiving myelosuppressive chemotherapy are required. Adapting this model as a training tool to provide physicians with personalized feedback and simplifying model output to be useful in point-of-care decision making could enhance physician education on FN risk assessment and appropriate utilization of G-CSF.

## Supporting Information

**Data S1.** Methods.

**Figure S1.** The predicted severe neutropenia (SN) risk or febrile neutropenia (FN) risk in the chemotherapy cycle 1 associated strongly with the actual FN risk in cycles 1–4 in the patient cohort on which the model was based.

**Figure S2.** Disposition of patients.

**Table S1.** Allowable chemotherapy regimens.

**Table S2.** Patient laboratory test results.

**Table S3.** Correlation between physician-assessed FN risk estimates and model–predicted risk estimates by patient-, physician-, and site-related characteristics.

**Table S4.** Correlation between physician-assessed FN risk estimates and G-CSF orders.

**Table S5.** Summary of physician-assessed risk FN estimates and model-predicted risk estimates for patients who received and did not receive orders for G-CSF.
