# Supplementary material for: Assessing patients’ risk of febrile neutropenia: is there a correlation between physician-assessed risk and model-predicted risk?
Source: Cancer Med. 2015 Mar 23;4(8):1153–60. doi: 10.1002/cam4.454 (PMC4559026; doi:10.1002/cam4.454)
Supplement: Supplementary file 1 [file cam40004-1153-sd1.doc]

SUPPLEMENTAL MATERIAL

METHODS

Determination of Sample Size

The sample size calculation assumed that FN risk assessments by the same physician are correlated between patients assessed by that physician. This correlation means that patients from the same physician have some duplicate (nonindependent) information. As a result of this correlation, larger sample sizes are needed than for a study with independent patients. The variance estimation procedures were modified to account for this correlation, and the sample size was determined by assuming some plausible correlation values. Investigators within a site were assumed to have a zero correlation. For sample size planning, a working model was used of

𝑌_𝑖𝑗_ = 𝛽_0_ + 𝛽_1_𝑋_𝑖𝑗_ + 𝜏_𝑖_ + 𝜖_𝑖𝑗_,

where 𝑋_𝑖𝑗_~𝑁(𝜇_𝑥_, 𝜎_𝑥_^2^ ) are the prediction tool FN risk probability scores for j^th^ patient of the i^th^ investigator, 𝜏_𝑖_~𝑁(0, 𝜎_𝜏_^2^ ) is a random intercept for the i^th^ investigator, 𝜖_𝑖𝑗_~𝑁(0, 𝜎_𝜖_^2^ ) is the error about the line, and the 𝑋_𝑖𝑗_, 𝜏_𝑖_, and 𝜖_𝑖𝑗_ are uncorrelated. Planning values of 𝜎_𝑥_ = 0.05, 𝜎_𝜖_ = 0.03, an intraphysician correlation of 0.3 (𝜎_𝝉_^2^/[𝜎_𝜖_^2^ + 𝜎_𝝉_^2^ ]), and an overall correlation of between FN risk probability scores of 0.3 were used. The calculation is robust to moderate departures from the planning standard deviations for 𝑋_𝑖𝑗_ and 𝜖_𝑖𝑗_. With 70 investigators and 14 subjects per investigator, the anticipated CI width was 0.117. The number of investigators was increased to 80 and the planned sample size to 1000 to allow for some potential exclusions or fewer subjects for some physicians. Because of lower than expected patient enrollment per investigator, 124 physicians were recruited to allow sufficient patient enrollment and adequate measures of precision.

Delete-a-Physician Jackknife Variance Estimator

A delete-a-physician (delete-a-cluster) jackknife is an estimation method for variances when the sample is a cluster sample. A typical jackknife works by removing one record from the data, recalculating the estimate of interest creating a replicate estimate, and then performing a weighted sum of squares of the differences between the replicate estimates and the overall estimate. When there is correlation between records (eg, between subjects reviewed by the same physician), the typical jackknife will tend to underestimate the sampling variance. To account for the correlation, the replicates are instead created by deleting all records for a physician at the same time. The resulting jackknife estimator ignores some within-physician variability but captures the between-physician variability. The jackknife also tends to slightly overestimate its target, leading to the estimate being close to the intended one, despite ignoring some of the within-physician variability.
