# Supplementary material for: Assessing patients’ risk of febrile neutropenia: is there a correlation between physician-assessed risk and model-predicted risk?
Source: Cancer Med. 2015 Mar 23;4(8):1153–60. doi: 10.1002/cam4.454 (PMC4559026; doi:10.1002/cam4.454)
Supplement: Supplementary file 2 [file cam40004-1153-sd2.doc]

Supplemental Figure 1. The predicted severe neutropenia (SN) risk or febrile neutropenia (FN) risk in the chemotherapy cycle 1 associated strongly with the actual FN risk in cycles 1–4 in the patient cohort on which the model was based.^14^
