# Supplementary material for: Assessing patients’ risk of febrile neutropenia: is there a correlation between physician-assessed risk and model-predicted risk?
Source: Cancer Med. 2015 Mar 23;4(8):1153–60. doi: 10.1002/cam4.454 (PMC4559026; doi:10.1002/cam4.454)
Supplement: Supplementary file 4 [file cam40004-1153-sd4.doc]

Supplemental Table 1. Allowable Chemotherapy Regimens

| **Chemotherapy Regimen** | **Frequency** |
| --- | --- |
| **Breast cancer** |  |
| TC | Every 21 days |
| Docetaxel 75 mg/m^2^, cyclophoshamide 600 mg/m^2^ |  |
| TCH | Every 21 days |
| Docetaxel 75 mg/m^2^, carboplatin AUC 5−7, trastuzumab dosed per institutional standards |  |
| AC | Every 21 days |
| Doxorubicin 60 mg/m^2^, cyclophosphamide 600 mg/m^2^ |  |
| AC + sequential taxane ± trastuzumab | Every 21 days |
| Doxorubicin 60 mg/m^2^, cyclophosphamide 600 mg/m^2^ |  |
| Docetaxel 100 mg/m^2^ or paclitaxel 175 mg/m^2^ |  |
| Trastuzumab dosed per institutional standards |  |
| CMF | Every 28 days |
| Cyclophosphamide 600 mg/m^2^/d × 2 nonsequential days, methotrexate 40 mg/m^2^/d × 2 nonsequential days, 5-fluorouracil 600 mg/m^2^/d × 2 nonsequential days |  |
| FEC ± sequential docetaxel | Every 21 days |
| 5-fluorouracil 500 mg/m^2^, epirubicin 100 mg/m^2^, cyclophosphamide 500 mg/m^2^ |  |
| Docetaxel 100 mg/m^2^ |  |
| **Non-small cell lung cancer** |  |
| Cisplatin, paclitaxel | Every 21 days |
| Cisplatin 75 mg/m^2^, paclitaxel 135 mg/m^2^ |  |
| Cisplatin, docetaxel | Every 21 days |
| Docetaxel 75 mg/m^2^, cisplatin 75 mg/m^2^ |  |
| Cisplatin, etoposide | Every 28 days |
| Cisplatin 100 mg/m^2^, etoposide 100 mg/m^2^/d × 3 days |  |
| Carobplatin, paclitaxel ± bevacizumab | Every 21 days |
| Paclitaxel 200 mg/m^2^, carboplatin AUC 5−7 (Calvert) |  |
| Bevacizumab ≤15 mg/kg |  |
| Carboplatin, docetaxel | Every 21 days |
| Docetaxel 75 mg/m^2^, carboplatin AUC 5−7 (Calvert) |  |
| **Small cell lung cancer** |  |
| CAV | Every 21−28 days |
| Cyclophosphamide 800 mg/m^2^, doxorubicin 50 mg/m^2^, vincristine 1.4−2.0 mg/m^2^ |  |
| Etoposide, carboplatin | Every 21−28 days |
| Etoposide 80−100 mg/m^2^/d × 3 days, carboplatin AUC 5−7 (Calvert) |  |
| Etoposide, cisplatin | Every 21−28 days |
| Etoposide 80−100 mg/m^2^/d × 3 days, cisplatin 100 mg/m^2^ |  |
| **Colorectal cancer** |  |
| FOLFOX4 ± bevacizumab | Every 14 days |
| 5-fluorouracil 400 mg/m^2^ bolus followed by 600 mg/m^2^ continuous infusion over 22 hours on days 1 and 2, leucovorin* 200 mg/m^2^ on days 1 and 2, oxaliplatin 85 mg/m^2^ on day 1 |  |
| Bevacizumab 5−10 mg/kg |  |
| FOLFOX6 ± bevacizumab | Every 14 days |
| 5-fluorouracil 400 mg/m^2^ bolus on day 1 followed by 1200 mg/m^2^ × 2 days (2400 mg/m^2^ over 46−48 hours starting on day 1), leucovorin* 400 mg/m^2^ on day 1, oxaliplatin 100 mg/m^2^ on day 1 |  |
| Bevacizumab 5−10 mg/kg |  |
| mFOLFOX6 ± bevacizumab | Every 14 days |
| 5-fluorouracil 400 mg/m^2^ bolus on day 1 followed by 1200 mg/m^2^ × 2 days (2400 mg/m^2^ over 46−48 hours starting on day 1), leucovorin* 400 mg/m^2^ on day 1, oxaliplatin 85 mg/m^2^ on day 1 |  |
| Bevacizumab 5−10 mg/kg |  |
| FOLFOX7 ± bevacizumab | Every 14 days |
| 5-fluorouracil (2400 mg/m^2^ over 46−48 hours starting on day 1), leucovorin* 400 mg/m^2^ on day 1, oxaliplatin 130 mg/m^2^ on day 1 |  |
| Bevacizumab 5−10 mg/kg |  |
| FOLFIRI ± bevacizumab | Every 14 days |
| 5-fluorouracil 400 mg/m^2^ bolus on day 1 followed by 1200 mg/m^2^ × 2 days (2400 mg/m^2^ over 46−48 hours starting on day 1), leucovorin* 400 mg/m^2^ on day 1, irinotecan 180 mg/m^2^ on day 1 |  |
| Bevacizumab 5−10 mg/kg |  |
| **Non-Hodgkin’s lymphoma** |  |
| CHOP | Every 21 days |
| Cyclophosphamide 750 mg/m^2^, doxorubicin 50 mg/m^2^, vincristine 1.4−2 mg/m^2^, prednisone dose per institutional standards |  |
| CHOP-R | Every 21 days |
| Cyclophosphamide 750 mg/m^2^, doxorubicin 50 mg/m^2^, vincristine 1.4−2 mg/m^2^, prednisone dose per institutional standards, rituximab dosed per institutional standards |  |
| CVP | Every 21−28 days |
| Cyclophosphamide 750 mg/m^2^, vincristine 1.4−2 mg/m^2^ |  |
| ± prednisone dosed per institutional standards |  |
| **Ovarian** |  |
| Carboplatin, docetaxel ± bevacizumab | Every 21 days |
| Carboplatin AUC 5−7 (Calvert), docetaxel 75 mg/m^2^ |  |
| Bevacizumab dosed per institutional standards |  |
| Carboplatin, paclitaxel ± bevacizumab | Every 28 days |
| Carboplatin AUC 2−4 (Calvert) × 3 nonsequential days, paclitaxel 80 mg/m^2^/d × 3 days |  |
| Bevacizumab dosed per institutional standards |  |

AC=cyclophosphamide, doxorubicin; AUC=area under the concentration-verus-time curve; CAV=cyclophosphamide, doxorubicin, vincristine); CHOP=cyclophosphamide, doxorubicin, vincristine, prednisone; CHOP-R=cyclophosphamide, doxorubicin, vincristine, prednisone, rituximab; CMF=cyclophosphamide, methotrexate, 5-fluorouracil; CVP=cyclophosphamide, vincristine, prednisone; FEC=5-fluorouracil, epiribucin, cyclophosphamide; FOLFIRI=fluorouracil, leucovorin, irinotecan; FOLFOX=fluorouracil, leucovorin, oxaliplatin; mFOLFOX=modified FOLFOX; TC=cyclophosphamide, docetaxel; TCH=carboplatin, docetaxel, trastuzumab.

*Levoleucovorin was allowed to be substituted for leucovorin at half of the stated dose.
