# Supplementary material for: Assessing patients’ risk of febrile neutropenia: is there a correlation between physician-assessed risk and model-predicted risk?
Source: Cancer Med. 2015 Mar 23;4(8):1153–60. doi: 10.1002/cam4.454 (PMC4559026; doi:10.1002/cam4.454)
Supplement: Supplementary file 5 [file cam40004-1153-sd5.doc]

Supplemental Table 2. Patient Laboratory Test Results*

|  | **Patients (N=944)** |
| --- | --- |
| Median (range) WBC, 10^9^/L | 7.4 (2.4−197.2) |
| Median (range) RBC, 10^12^/L | 4.3 (2.1−6.1) |
| Median (range) platelets, 10^9^/L | 268.0 (56.0−938.0) |
| Median (range) serum creatinine, µmol/L | 70.7 (35.3−742.6) |
| Median (range) GFR, mL/min | 88.6 (7.7−252.7) |
| Elevated AST, n (%) | 99 (10) |
| Elevated bilirubin, n (%) | 18 (2) |
| Elevated alkaline phosphatase, n (%) | 136 (14) |
| Elevated serum albumin, n (%) | 3 (<1) |
| Elevated BUN, n (%) | 119 (13) |

AST=aspartate aminotransferase; BUN=blood urea nitrogen; GFR=glomerular filtration rate; RBC=red blood cell; WBC=white blood cell.

*Primary analysis set.
