# Supplementary material for: Assessing patients’ risk of febrile neutropenia: is there a correlation between physician-assessed risk and model-predicted risk?
Source: Cancer Med. 2015 Mar 23;4(8):1153–60. doi: 10.1002/cam4.454 (PMC4559026; doi:10.1002/cam4.454)
Supplement: Supplementary file 6 [file cam40004-1153-sd6.doc]

**Supplemental Table 3. Correlation Between Physician-Assessed FN Risk Estimates and Model–Predicted Risk Estimates by Patient-, Physician-, and Site-Related** Characteristics

| **Characteristic** | **n** | **Correlation Estimate* (95% CI^†^)** |
| --- | --- | --- |
|  | 944 | 0.249 (0.179–0.316) |
| Patient-related characteristic |  |  |
| Patient age |  |  |
| Age <65 years | 533 | 0.263 (0.155 to 0.364) |
| Age ≥65 years | 391 | 0.227 (0.122 to 0.328) |
| Tumor type |  |  |
| Breast | 364 | 0.166 (0.027 to 0.298) |
| Colorectal | 259 | 0.130 (−0.068 to 0.318) |
| Non-small cell lung | 115 | 0.121 (−0.134 to 0.362) |
| Non-Hodgkin’s lymphoma | 106 | 0.194 (0.000 to 0.373) |
| Small cell lung | 83 | 0.008 (−0.309 to 0.323) |
| Ovarian | 17 | −0.050 (−0.483 to 0.403) |
| Planned chemotherapy regimen of interest |  |  |
| TC | 198 | 0.189 (0.014 to 0.354) |
| FOLFOX | 218 | 0.082 (−0.057 to 0.218) |
| CHOP-based | 101 | 0.115 (−0.093 to 0.313) |
| TCH | 83 | 0.254 (−0.045 to 0.511) |
| AC | 49 | −0.185 (−0.581 to 0.283) |
| AC+T | 22 | −0.150 (−0.486 to 0.224) |
| Physician-related characteristic |  |  |
| Physician years in clinical practice |  |  |
| ≤8 years of practice | 340 | 0.175 (0.046 to 0.299) |
| >8−17 years of practice | 305 | 0.306 (0.171 to 0.429) |
| >17 years of practice | 299 | 0.255 (0.129 to 0.373) |
| Primary specialty |  |  |
| Oncologist | 198 | 0.286 (0.169 to 0.395) |
| Hematologist/oncologist | 743 | 0.239(0.154 to 0.321) |
| Gynecologist/oncologist | 3 | −0.096 (NE) |
| Site-related characteristic |  |  |
| Mean number of patients treated per month |  |  |
| ≤212 patients | 342 | 0.306 (0.162 to 0.436) |
| >212−415 patients | 304 | 0.259 (0.117 to 0.391) |
| >415 patients | 298 | 0.180 (0.033 to 0.319) |
| Type of clinical practice |  |  |
| Single specialty | 615 | 0.247 (0.158 to 0.333) |
| Multiple subspecialties | 329 | 0.262 (0.099 to 0.412) |
| Clinical setting |  |  |
| ≤4 physicians | 655 | 0.247 (0.160 to 0.331) |
| >4 physicians | 289 | 0.253 (0.130 to 0.369) |

AC=cyclophosphamide, doxorubicin; AC+T=AC + sequential taxane ± trastuzumab; CHOP=cyclophosphamide, doxorubicin, vincristine, prednisone; FN=febrile neutropenia;FOLFOX=fluorouracil, leucovorin, oxaliplatin; NE=not estimated; SN=severe neutropenia; TC=cyclophosphamide, docetaxel; TCH=carboplatin, docetaxel, trastuzumab.

*Correlations can range from 1 (perfect correlation) to −1, where 0 is no correlation, and negative correlations represent inverse relationships.

^†^Approximate CI computed using the cluster jackknife estimator and Wald method utilizing Fisher transformation.
