# Supplementary material for: Assessing patients’ risk of febrile neutropenia: is there a correlation between physician-assessed risk and model-predicted risk?
Source: Cancer Med. 2015 Mar 23;4(8):1153–60. doi: 10.1002/cam4.454 (PMC4559026; doi:10.1002/cam4.454)
Supplement: Supplementary file 7 [file cam40004-1153-sd7.doc]

**Supplemental Table 4. Correlation Between Physician-Assessed FN Risk Estimates and G-CSF Orders**

| **Characteristic** | **n** | **Correlation Estimate* (95% CI^†^)** |
| --- | --- | --- |
|  | 944 | 0.313 (0.135–0.472) |
| Patient age |  |  |
| Age <65 years | 553 | 0.307(0.076 to 0.507) |
| Age ≥65 years | 391 | 0.318 (0.164 to 0.457) |
| Tumor type |  |  |
| Breast | 364 | 0.150 (−0.086 to 0.370) |
| Colorectal | 259 | 0.514 (0.384 to 0.625) |
| Non-small cell lung | 115 | 0.265 (−0.026 to 0.514) |
| Non-Hodgkin’s lymphoma | 106 | 0.097 (−0.368 to 0.524) |
| Small cell lung | 83 | 0.161 (−0.138 to 0.434) |
| Ovarian | 17 | 0.359 (−0.082 to 0.682) |
| Planned chemotherapy regimen of interest |  |  |
| TC | 198 | 0.255 (0.081 to 0.414) |
| FOLFOX | 218 | 0.478 (0.293 to 0.628) |
| CHOP-based | 101 | 0.011 (−0.484 to 0.500) |
| TCH | 83 | −0.007 (−0.273 to 0.260) |
| AC | 49 | −0.170 (−0.512 to 0.218) |
| AC+T | 22 | 0.311 (−0.562 to 0.857) |
| Physician years in clinical practice |  |  |
| ≤8 years of practice | 340 | 0.403 (0.266 to 0.524) |
| >8−17 years of practice | 305 | 0.451 (0.322 to 0.564) |
| >17 years of practice | 299 | 0.145 (−0.230 to 0.482) |
| Primary specialty |  |  |
| Oncologist | 198 | 0.452 (0.318 to 0.568) |
| Hematologist/oncologist | 743 | 0.284 (0.075 to 0.469) |
| Gynecologist/oncologist | 3 | 1.000 (NE) |
| Mean number of patients treated per month |  |  |
| ≤212 patients | 342 | 0.477 (0.385 to 0.560) |
| >212−415 patients | 304 | 0.140 (−0.216 to 0.463) |
| >415 patients | 298 | 0.394 (0.255 to 0.517) |
| Type of clinical practice |  |  |
| Single specialty | 615 | 0.310 (0.051 to 0.530) |
| Multiple subspecialties | 329 | 0.331 (0.151 to 0.491) |
| Clinical setting |  |  |
| ≤4 physicians | 655 | 0.278 (0.047 to 0.480) |
| >4 physicians | 289 | 0.422 (0.273 to 0.551) |

AC=cyclophosphamide, doxorubicin; AC+T=AC + sequential taxane ± trastuzumab; CHOP=cyclophosphamide, doxorubicin, vincristine, prednisone; FN=febrile neutropenia; FOLFOX=fluorouracil, leucovorin, oxaliplatin; G-CSF=granulocyte colony-stimulating factor; NE=not estimated; TC=cyclophosphamide, docetaxel; TCH=carboplatin, docetaxel, trastuzumab.

*Correlations can range from 1 (perfect correlation) to −1, where 0 is no correlation, and negative correlations represent inverse relationships.

^†^Approximate CI computed using the cluster jackknife estimator and Wald method utilizing Fisher transformation.
