# Supplementary material for: Assessing patients’ risk of febrile neutropenia: is there a correlation between physician-assessed risk and model-predicted risk?
Source: Cancer Med. 2015 Mar 23;4(8):1153–60. doi: 10.1002/cam4.454 (PMC4559026; doi:10.1002/cam4.454)
Supplement: Supplementary file 8 [file cam40004-1153-sd8.doc]

Supplemental Table 5. Summary of Physician-Assessed Risk FN Estimates and Model-Predicted Risk Estimates for Patients Who Recieved and Did Not Receive Orders for G-CSF*

|  | **Received G-CSF Order (n=634)** | **Did Not Receive G-CSF Order (n=310)** | **Total (N=944)** |
| --- | --- | --- | --- |
| Median (Q1−Q3) physician-assessed FN risk estimate over all chemotherapy cycles, % | 25.0 (20.0–35.0) | 15.0 (10.0–20.0) | 20.0 (15.0–30.0) |
| Median (Q1−Q3) model-predicted risk estimate, % | 22.2 (9.1–39.7) | 8.5 (4.1–24.8) | 17.9 (6.9−35.8) |
| Correlation estimate (approximate 95% CI^†^) | 0.172^‡^ (0.088–0.254) | 0.239^‡^ (−0.000–0.453) | 0.249^‡^ (0.179−0.316) |

FN=febrile neutropenia; G-CSF=granulocyte colony-stimulating factor; SN=severe neutropenia.

*Primary analysis set.

^†^Confidence interval calculated using the cluster jackknife estimator and Wald method with Fisher transformation.

^‡^Correlations can range from 1 (perfect correlation) to −1, where 0 is no correlation, and negative correlations represent inverse relationships.
